# Role of MTHFR 677C>T and 1298A>C gene polymorphisms on renal toxicity caused by lead exposure in wastewater treatment plant workers

**DOI:** 10.1007/s11356-023-28309-y

**Published:** 2023-06-28

**Authors:** Amal Saad-Hussein, Wafaa Ghoneim Shousha, Sara Yahya Mohamed Al-Sadek, Shimaa Shawki Ramadan

**Affiliations:** 1 Environmental and Occupational Medicine Department, Environment and Climate Change Research Institute, National Research Centre, Cairo, Egypt; 2 Biochemistry division, Chemistry Department, Faculty of Science, Helwan University, Cairo, Egypt

**Keywords:** Lead, Renal toxicity, *MTHFR* gene polymorphisms, Homocysteine, Oxidative-antioxidant

## Abstract

Environmental and occupational lead (Pb) exposures continue to pose major public health problems. Wastewater treatment plant (WWTP) workers proved are exposing to high Pb concentrations in sludge departments. The aim of the work was to investigate the role of *MTHFR C677T* and *MTHFR A1298C* gene polymorphisms on alteration of oxidative stress and homocysteine levels in WWTP workers exposed to high Pb concentrations, and study its relations with renal functions. The study included 90 WWTP workers from Abu-Rawash WWTP. Homocysteine, creatinine, urea, malondialdehyde (MDA), and total antioxidant capacity (TAC) were measured. Polymorphisms of *MTHFR C677CT* and *MTHFR A1298C* genes were studied using PCR/RFLP. Urine Pb concentrations were also measured. About 32.2% of the workers were with detectable Pb levels. Pb, homocysteine, and MDA levels were significantly higher among workers carrying *TT* polymorphism compared to other *MTHFR C677T* gene polymorphisms, while TAC was significantly lower among them compared to other polymorphisms. The same results were found among workers carrying *CC* compared to other *MTHFR A1298C* gene polymorphisms. WWTP workers carrying *MTHFR 677TT* and *MTHFR 1298CC* are more susceptible to elevation of homocysteine and the urinary Pb compared to the workers with the other polymorphisms. Furthermore, those workers were found to have increase in urea and creatinine. Therefore, *MTHFR C677T* and *MTHFR A1298C* gene polymorphisms could be used for prediction of the susceptibility to the risk of kidney impairments among WWTP workers in the sludge departments caused by their exposure to high Pb in their workplace.

## Introduction

Lead (Pb) is a worldwide environmental contaminant. Exposure to Pb occurs through ingestion, inhalation, and skin contact via water, food, air, and soil. Pb is mainly accumulated in the liver and kidney (Gundacker et al. 2021) and is primarily eliminated through urine and feces (ATSDR 2017). Pb potentially induces oxidative stress, which has a role in the pathophysiology of induction of tissue damage of the brain, kidney, liver, heart, reproductive organs, and erythrocytes (Shih et al. 2007). Thus, the damaging mechanism of Pb could be mainly attributed to oxidative stress induction (Seven et al. 2021).

Peroxidation of lipid is thought to be the most valid marker of oxidative stress (Thakur et al. 2017). Lipid peroxidation has many fine products; malondialdehyde (MDA) is the most important and the most commonly known oxidative stress biomarker (Gaweł et al. 2004). The highly reactive thiol group of homocysteine can undergo rapid auto-oxidization in the presence of oxygen and lead-generating potent reactive oxygen species such as hydrogen peroxide and superoxide anion (Caylak et al. 2008). An increase in homocysteine levels is considered a risk factor for vascular diseases as it causes damage to vascular endothelial functions (Balint et al. 2020). Kidneys are very sensitive to vascular endothelial impairment, and vascular endothelium damage occurs early in chronic kidney diseases (Roumeliotis et al. 2020)

The methylenetetrahydrofolate reductase (MTHFR) is a key enzyme in the homocysteine metabolism that stimulates the conversion of 5,10-methylenetetrahydrofolate to 5-methyltetrahydrofolate, the major circulating folate form (Gupta et al. 2019). Normal MTHFR activity is required to prevent homocysteine accumulation (Yakub et al. 2017). Moreover, the polymorphism of *MTHFR* gene was found to have a protective role on renal function as proved by Trovato et al. (2015). They found that MTHFR 677C>T and A1298A>C gene polymorphisms could have a protection from complicated end-stage renal failure among the included dialysis patients.

Saad-Hussein (2020) found that sewage workers in Abu-Rawash wastewater treatment plant (WWTP) were exposing to high Pb concentrations during the processes of wastewater treatment in the sludge departments. About 13% of the exposed workers were complaining of renal abnormal symptoms. Therefore, further studies were suggested to investigate the association between exposure to high concentration of Pb in sludge department of the WWTPs on the kidney functions of the exposed workers and to find an early predictor method to minimize the economic and social burden of kidney failure among the vulnerable workers Okpogba et al. 2021.

This study aimed to explore the role of *MTHFR C677T* and *MTHFR A1298C* gene polymorphisms on alteration of oxidative stress and homocysteine levels in wastewater treatment plant (WWTP) workers exposed to high Pb concentrations and study its relations with renal functions.

## Subjects and methods

The study was a cross-sectional cohort study without control group. About 90 workers from Abu-Rawash WWTP were included in the present study, after exclusion of the smokers and those employed for less than 10 years. All the included workers were selected within the age range 35–55 years, from the sludge departments that proved to have high Pb concentrations in the workplace, as Pb was found to be about 50.1 ± 38.4 mg/kg sludge (Saad-Hussein 2020).

Ethical approval was obtained from the Ethical Committee of National Research Center. Written consents were also obtained from all the enrolled persons.

### Samples collection

Five milliliters of venous blood sample was taken from each subject during their working shifts and partitioned into two tubes. Two milliliters of blood was placed in an EDTA tube and stored at – 20 °C for studying of *MTHFR C677T* and *MTHFR A1298C* polymorphisms. The remaining 3 ml of blood sample was retained in a sterile tube, allowed to clot for 30 min at 37 °C, then centrifuged at 3000 rpm for 10 min, and the serum was kept frozen at – 20 °C. TAC, MDA, homocysteine, creatinine, and urea were measured in the serum.

### Measurement of serum total antioxidant capacity (TAC)

Serum antioxidants are reacted with a predefined quantity of exogenously provided hydrogen peroxide to assess total antioxidant capacity (TAC) (H_2_O_2_). The sample’s antioxidants remove some of the H_2_O_2_ provided, and the remaining H_2_O_2_ is then colorimetrically detected through an enzymatic reaction that results in a colored product which is then measured at 505 nm (Koracevic et al. 2001).

### Measurement of serum malondialdehyde (MDA) level

Thiobarbituric acid reactive species (TBARS), a pink substance, was measured to assess malondialdehyde (MDA). TBARS were measured calorimetrically at 530 nm (Yagi 1982).

Creatinine and urea were measured in blood using spectrophotometric method (Diamond kit, Egypt).

Homocysteine level was estimated in blood via enzyme-linked immunosorbent assay (ELK Biotechnology, China).

### Genotyping of MTHFR C677T and MTHFR A1298C polymorphisms

Genotypes of the *MTHFR C677T* and *MTHFR A1298C* polymorphisms were investigated by PCR/RFLP. DNA was extracted from the whole blood samples by using the genomic DNA extraction kit (Gene JET™/Fermentas). Five microliters of extracted DNA, 12.5 μl of master mix buffer (2X), 2 μl of (0.4 μM) forward primer, 2 μl of (0.4 μM) reverse primer, and water (nuclease-free) were added to make the final volume 25 μl. The thermal cycler was adjusted for 30 cycles of denaturation at 94 °C for 30 s, annealing at 55 °C for 45 s, extension at 72 °C for 1 min, the final cycle was followed by elongation at 72 °C for 5 min. The region responsible for the *MTHFR C677T* polymorphism was amplified using the following primers:

- Forward primer—5′-TGAAGGAGAAGGTGTCTGCGGGA-3′
- Backward primer—5′-AGGACGGTGCGGTGAGAGTG-3′

After RFLP analyses using Hinf 1 enzyme in 37 °C for 1 h, DNA fragments were visualized in a 2% ethidium bromide–stained agarose gel and photographed under UV light to determine *MTHFR* genotypes: *TT* (175, 23 bp), *CT* (198, 175, and 23 bp), and *CC* (198 bp).

The region responsible for the *MTHFR A1298C* polymorphism was amplified using the following primers:

- Forward primer—5′-CTTTGGGGAGCTGAAGGACTACTAC-3′
- Backward primer—5′-CACTTTGTGACCATTCCGGTTTG-3′

After RFLP analyses using Mbo II enzyme, DNA fragments were visualized in a 2% ethidium bromide–stained agarose gel and photographed under UV light to determine *MTHFR* genotypes: *AA* (56, 31, 30, 28, and 18 bp), *AC* (84, 56, 31, 30, 28, and 18 bp) and *CC* (84, 31, 30, and 18 bp) (Amarakoon and Fernandopulle 2016).

Lead (Pb) in urine was determined by using the Agilent 5100 Synchronous Vertical Dual View (SVDV) ICP-OES, with Agilent Vapor Generation Accessory VGA 77. Urine samples were digested to have suitable matrix for measuring of the metal ions and to provide acceptable and consistent recovery suitable with the analytical method (APHA et al. 2017).

### Statistical analysis

Statistical analysis was performed using SPSS version 23 for the collected data and the laboratory results. Quantitative results were expressed as mean ± SD (standard deviation). Pearson correlation was used to define the relation between the quantitative variables. ANOVA was used to compare quantitative results between more than two groups, and post hoc least significant difference (LSD) was used to compare between each two independent groups. Kruskal-Wallis test was used for comparing quantitative variables with Skewness. Significance levels were set at *P* ≤ 0.05. Tabulation and figures were done using excel program.

## Results

The urinary Pb concentration of the included workers were ranged between undetectable values (< 0.1 μg/mg) and 20.59 μg/mg (1.33±0.35 μg/mg). About 32.2% of the workers were with detectable Pb levels.

There were positive significant correlations between the urinary Pb concentrations with creatinine, urea, and homocysteine and between MDA levels and creatinine and urea levels, while there was no significant relationships between TAC and the Pb concentration, creatinine, and urea (Table 1).

**Table 1:** Relationships between the levels of urinary Pb, homocysteine, TAC, and MDA, and the levels of urinary Pb, creatinine, and urea

		Pb (μg/mg)	Homocysteine (μmol/L)	TAC	MDA
Pb (μg/mg)	*r*=	1	0.4^*^	− 0.3	0.2
	Sig. (2-tailed)		0.05	0.2	0.3
Creatinine (mg/dl)	r=	0.4^*^	0.03	0.3	0.4*
	Sig. (2-tailed)	0.02	0.8	0.2	0.05
Urea (mg/dl)	*r*=	0.5^**^	0.2	0.2	0.4*
	Sig. (2-tailed)	0.007	0.3	0.4	0.05

*P < 0.05, **P < 0.005

**Fig. 1:**
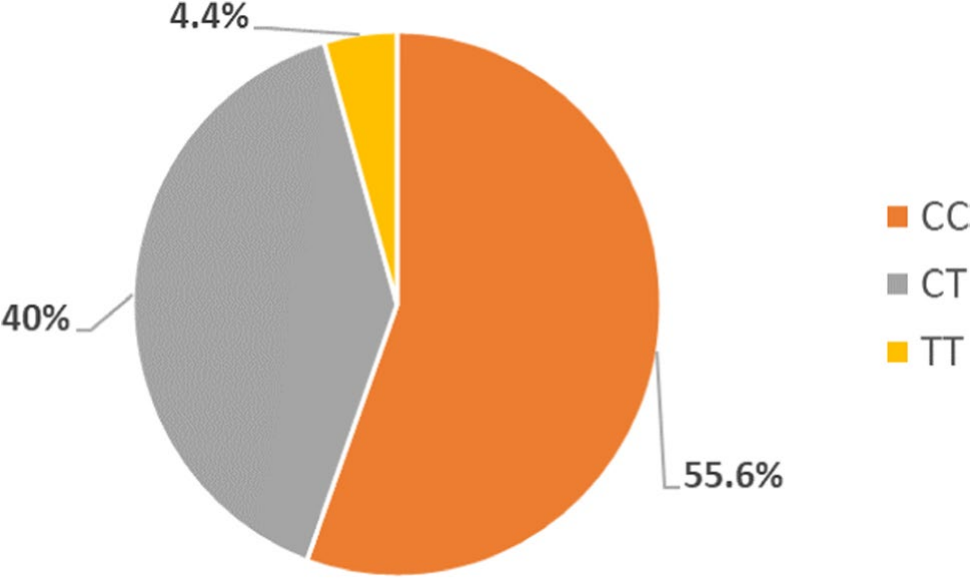
Distribution of *MTHFR C677T* gene polymorphism among the workers

Figure 1 shows that *CC* polymorphism of the *MTHFR C677T* gene was the highest polymorphism among the examined workers, and *TT* was the least polymorphism.

Urinary Pb concentration, homocysteine, and MDA were significantly higher in workers with TT polymorphism compared to workers with the other polymorphisms. Homocysteine was also significantly higher in workers with CT allele than in workers with CC alleles. TAC was significantly lower in workers with TT polymorphism compared to the workers with the other polymorphisms, while there were no significant differences between the workers with the different alleles as regards creatinine and urea (Table 2).

**Table 2:** Comparisons of the urinary Pb, kidney functions, and oxidative-antioxidant biomarkers levels between the different polymorphism of C677T MTHFR gene

	C677T MTHFR gene polymorphism						ANOVA	
	CC (50)		CT (36)		TT (4)			
	Mean	SD	Mean	SD	Mean	SD	*F* ratio	*P* value
Pb (μg /mg)	0.85	0.28	1.30	0.54	7.55	1.90	8.579	<0.0001***
	(TT)		(TT)		(CC,CT)			
Creatinine (mg/dl)	0.97	0.06	0.96	0.03	0.98	0.03	.150	0.861
Urea (mg/dl)	32.02	.97	31.53	.96	27.75	2.46	.832	0.439
Homocysteine conc. (μmol/L)	12.75	.99	31.40	2.00	46.56	1.05	57.550	<0.0001***
	(CT,TT)		(CC,TT)		(CC,CT)			
TAC. (mM/L)	0.88	0.10	1.07	0.15	0.22	0.01	3.299	0.04*
	(TT)		(TT)		(CC.CT)			
MDA (nmol/ml)	9.72	0.87	9.92	1.25	17.12	0.01	3.536	0.03*
	(TT)		(TT)		(CC,CT)			

*P < 0.05, ***P < 0.0001

**Fig. 2:**
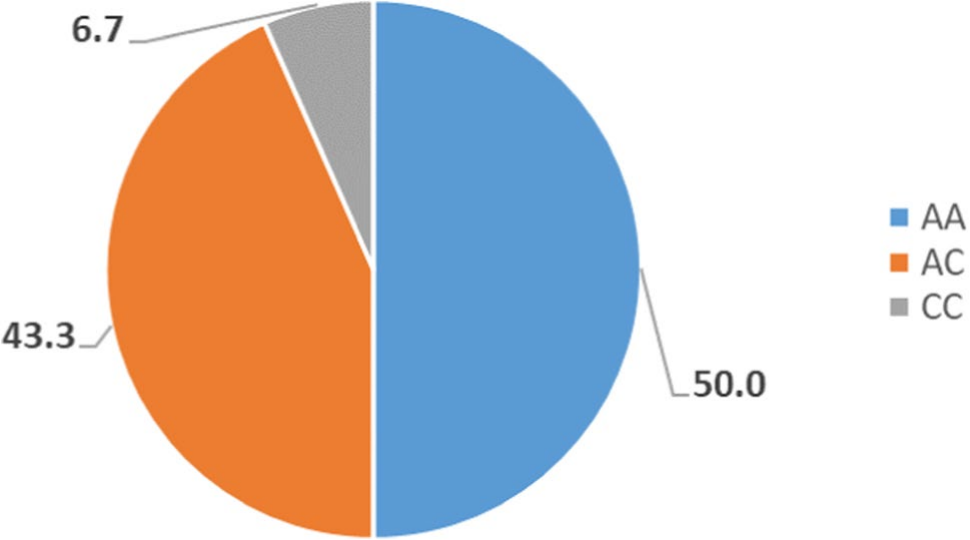
Distribution of *MTHFR A1298C* gene polymorphism among the workers

Figure 2 shows that *AA* polymorphism of the *MTHFR A1298C* gene was the highest polymorphism among the examined workers, and *CC* polymorphism was the least polymorphism.

Urinary Pb concentration and homocysteine were significantly higher in workers with CC polymorphism compared to workers with the other polymorphisms. Homocysteine was also significantly higher in workers with AC alleles than the workers with AA alleles. Regarding MDA, although there was no significant difference between the workers with the different polymorphisms by ANOVA, but on comparing each two groups, a significant increase in MDA was detected in the workers with CC polymorphism compared to the workers with AC and of AA. There were no significant differences between studied groups as regards creatinine and urea (Table 3).

**Table 3:** Comparisons of urinary Pb, kidney functions, and oxidative-antioxidant biomarkers levels between the workers with different polymorphism of A1298C MTHFR gene

	A1298C MTHFR gene polymorphism						ANOVA	
	AA (45)		AC (39)		CC (6)			
	Mean	SD	Mean	SD	Mean	SD	*F* ratio	*P* value
Pb (μg/mg)	0.82	0.30	1.20	0.51	5.98	3.26	5.761*	0.05
	(CC)		(CC)		(AA,AC)			
Creatinine (mg/dl)	0.94	0.07	0.94	0.03	1.00	0.03	1.253	0.291
Urea (mg/dl)	33.00	0.96	30.67	0.99	27.67	2.03	2.735	0.07
Homocystein conc (μmol/L)	12.84	1.02	28.20	2.09	46.14	1.09	44.643	<0.0001
	(AC,CC)		(AA,CC)		(AA,AC)			
TAC (mM/L)	0.95	0.11	0.93	0.13	0.55	0.35	1.776*	0.411
MDA (nmol/ml)	9.95	0.96	9.58	1.06	15.78	2.50	2.885	0.064
	(CC)		(CC)		(AA,AC)			

*Kruskal-Wallis test was used

## Discussion

Workers in WWTP and farmers using sludge as fertilizer were proved to have higher concentrations of lead (Pb) in their biological fluids (Ibrahem et al. 2020). The present study revealed that about 32.2% of the workers were with detectable Pb levels. Normally Pb must be below the detectable levels (< 0.1 μg/mg).

Ngozyka et al. (2021) reported that there was significant increase in creatinine and urea in workers exposed to heavy metals at work due to impairment in their renal function. Both creatinine and urea are indicators of kidney function and are continually maintained within a reference limit in healthy patients (Allen 2012). In the present study, there was positive correlations between the urinary Pb concentrations with the creatinine and urea levels of the workers.

Homocysteine and MDA are sensitive to Pb toxicity (Kasperczyk et al. 2017). Elevation of the levels of homocysteine are likely to be consequence of heart failure–related renal insufficiency (Schofield et al. 2003). In the present study, homocysteine was significantly correlated with MDA levels, and there were positive correlations between MDA levels and creatinine and urea level concentrations in the included workers. Therefore, homocysteine and MDA were found to have significant roles in affecting creatinine and urea among the workers occupationally exposed to Pb and could be considered as a risk factor to the development kidney function impairments. That was also proved by the significant positive correlations between the urinary Pb concentrations with the creatinine and urea levels in the included workers in the present study.

In the present study, roles of *MTHFR 677C>T* and *MTHFR 1298A>C* gene polymorphisms on altered oxidative-antioxidant relationship were investigated in WWTP workers exposed to high Pb concentrations and were studied in relations to the renal functions of the workers.

The distribution of *MTHFR C677T* genotypic among the included workers were *CC* (55.6%), *CT* (40%), and *TT* (4.4%), and the frequencies of *MTHFR A1298C* genotypic were *AA* (50.0%), *AC* (43.3%), and *CC* (6.7%).

Ramos-Esquivel et al. (2020) detected that *MTHFR C677T* genotypic frequencies were distributed as follows: *CC* (30%), *TT* (20%), and *CT* (50%), and *MTHFR A1298C* genotypic frequencies were *AA* (70%), *CC* (4%), and *AC* (26%). Therefore, the present study revealed that the distribution of *MTHFR A1298C* genotypic seemed to be similar distributed to that detected by Ramos et al. study, but not in the same percentages, while the distribution of *MTHFR C677T* genotypic in the present study was different from that in Ramos et al. study.

Various studies had revealed that the *MTHFR C677T* gene polymorphism is associated with elevated level of homocysteine, and that *T* allele carrier are with higher risk for hyper-homocysteinemia compared to *C* allele (Wu et al. 2014; Bagheri Hamidi et al. 2020. It was suggested that this variation may be a possible genetic predisposing factor for renal injury development in Egyptian hypertensive subjects (Elsaid et al. 2021), while Pramukarso et al. (2015) disagreed with these findings, as they found that *CC* alleles associated with the development of hyper-homocysteinemia.

In the present study, homocysteine and MDA were found to be significantly elevated in workers carrying of *TT* genotype of the *MTHFR C677T* gene compared to those carrying the other two genotypes. There was also significant reduction in TAC in the workers carrying *TT* compared to the other genotypes. In addition, homocysteine of the workers carrying *CT* genotype was found to be also significantly higher compared to those carrying *CC* genotype. This was with the finding of both Wu et al. (2014) and Bagheri Hamidi et al. (2020) and could be due to that *T* allele carriers are at higher risk for hyper-homocysteinemia compared to those carrying *C* allele. So, the elevation of MDA and homocysteine and the reduction in TAC could explain the significant elevation of the urinary Pb in the workers carrying *TT* genotype compared to the workers with the other two genotypes. These significant elevations may result in the elevation of the urea and creatinine in the workers carrying *TT* genotype of the *MTHFR C677T* gene. This was with the results of Trovato et al. (2015), as they detected that *MTHFR C677T* mutation (*TT*) was with kidney involvement. Shen et al. (2015) found that *MTHFR* gene polymorphisms are correlated with bone mineral loss, which is a main endogenous Pb source, and this may explain the elevation of urinary Pb in workers carrying *TT* genotype of the *MTHFR C677T* gene compared to those carrying the other two genotypes.

Similarly, Ramos-Esquivel et al. (2020) found a statistical significant difference in the occurrence of severe anemia and thrombocytopenia among patients with at least one *C* mutant allele of the *MTHFR A1298C* polymorphism in comparison with wild-type individuals. The *MTHFR 1298CC* mutant-type homozygote carriers were found to be exhibited higher blood Pb concentration than the *1298AC/AA* carriers (Shen et al. 2015). They attributed the relationship of blood Pb elevation among *CC* genotype of *MTHFR A1298C* gene due to the reduction in the *MTHFR* enzyme activity and the consequential elevation of the homocysteine levels.

In agreement to the previous publications, the present study revealed that urinary Pb concentration and homocysteine levels were significantly higher in workers carrying *CC* genotype of the *MTHFR A1298C* gene. This significant elevation in the Pb and homocysteine could attribute to the slight elevation in the kidney function biomarkers; urea, and creatinine.

## Conclusion

The WWTP workers carrying *MTHFR 677TT* as well as *MTHFR 1298CC* are more vulnerable to elevation of the Pb excretion in their urine, and elevation in the homocysteine levels than the workers carrying *MTHFR 677CC/CT* or *MTHFR 1298AA/AC*, respectively. These elevations in Pb and homocysteine, as well as MDA, could lead to elevation in the urea and creatinine among them. Therefore, the polymorphisms of these two genes could be used as predictor gene susceptibility to kidney impairments among those sludge exposed workers.

## Data Availability

The data is available if it is requested.
